# Development of a Fetal Weight Chart Using Serial Trans-Abdominal Ultrasound in an East African Population: A Longitudinal Observational Study

**DOI:** 10.1371/journal.pone.0044773

**Published:** 2012-09-21

**Authors:** Christentze Schmiegelow, Thomas Scheike, Mayke Oesterholt, Daniel Minja, Caroline Pehrson, Pamela Magistrado, Martha Lemnge, Vibeke Rasch, John Lusingu, Thor G. Theander, Birgitte Bruun Nielsen

**Affiliations:** 1 Centre for Medical Parasitology, Institute of International Health, Immunology, and Microbiology, University of Copenhagen and Department of Infectious Diseases, Copenhagen University Hospital, Copenhagen, Denmark; 2 Department of Biostatistics, Institute of Public Health, University of Copenhagen, Copenhagen, Denmark; 3 Department of Medical Microbiology, Radboud University Nijmegen Medical Centre, Nijmegen, The Netherlands; 4 Tanga Medical Research Center, National Institute for Medical Research, Tanga, Tanzania; 5 Department of Clinical Pathology, Odense University Hospital, Odense, Denmark; 6 Department of Obstetrics and Gynecology, Odense University Hospital, Odense, Denmark; 7 Department of Obstetrics and Gynecology, Aarhus University Hospital, Skejby, Denmark; Assiut University Hospital, Egypt

## Abstract

**Objective:**

To produce a fetal weight chart representative of a Tanzanian population, and compare it to weight charts from Sub-Saharan Africa and the developed world.

**Methods:**

A longitudinal observational study in Northeastern Tanzania. Pregnant women were followed throughout pregnancy with serial trans-abdominal ultrasound. All pregnancies with pathology were excluded and a chart representing the optimal growth potential was developed using fetal weights and birth weights. The weight chart was compared to a chart from Congo, a chart representing a white population, and a chart representing a white population but adapted to the study population. The prevalence of SGA was assessed using all four charts.

**Results:**

A total of 2193 weight measurements from 583 fetuses/newborns were included in the fetal weight chart. Our chart had lower percentiles than all the other charts. Most importantly, in the end of pregnancy, the 10^th^ percentiles deviated substantially causing an overestimation of the true prevalence of SGA newborns if our chart had not been used.

**Conclusions:**

We developed a weight chart representative for a Tanzanian population and provide evidence for the necessity of developing regional specific weight charts for correct identification of SGA. Our weight chart is an important tool that can be used for clinical risk assessments of newborns and for evaluating the effect of intrauterine exposures on fetal and newborn weight.

## Introduction

Small for gestational age (SGA) and intrauterine growth restricted infants have an increased risk of mortality and morbidity [1]–[4]. SGA is often used as a proxy for intrauterine growth restriction and defined as a weight below the 10^th^ percentile on a population based weight chart [3].

Very few weight charts have been produced on African populations, and the charts that do exist are mostly based on birth weights (BW) [5], [6]. Charts developed from BWs have substantial lower percentiles compared to charts based on fetal weights (FW) [7]. This is caused by a higher prevalence of intrauterine growth retardation among preterm deliveries and the preterm newborn therefore tends to be smaller than the unborn fetus, hereby lowered the percentiles of BW charts [8]. The lower percentiles can result in under-diagnosing SGA [9].

In Africa, FW charts only exist for Kinshasa, Congo and Burkina Faso, and they both differ from charts for white populations [10], [11]. Studies have shown that optimal BW [12], FW [10], fetal biometric measurements [13]–[16] and possibly end-pregnancy fetal growth velocity [17], [18] differ between geographical areas and ethnic groups. The ethnic groups of Congo and Burkina Faso are different from Tanzania and the geographical distance from northeastern Tanzania to Kinshasa, Congo as well as to Burkina Faso is considerable. It could, therefore, be questioned whether the mentioned charts are appropriate in Tanzania. Furthermore, weight charts could be improved by excluding pathological pregnancies, since pathological pregnancies lead to a lowered mean FW and BW and cause an underestimation of the prevalence of SGA [19], [20]. Adjustment for pathology was not done in the mentioned charts.

The objective of this study was to produce a new weight chart for a Tanzanian population using gestational age and FWs from longitudinal ultrasound investigations and BWs from healthy pregnancies. We compared this chart to the Congolese chart [10] and the Hadlock chart representing a white population [21].

Mikolajczyk *et al* [20] suggested to adjust the Hadlock chart [21] using country specific mean BWs, when ultrasound derived weight charts are not available for a given population. Our data allowed for the first time in an African population to compare the performance of the method by Mikolajczyk *et al* [20] to a ultrasound derived chart. Our data indicate that the modified Hadlock chart better reflected the local situation than the other charts used for comparison.

## Methods

### Ethics statement

The study received ethical approval from the Tanzania Medical Research Coordinating Committee (MRCC) on the 18^th^ of April 2008 with reference number NIMR7HQ/R.8a/Vol. IX/688. MRCC is the National Regulatory Body responsible for the supervision of health research and ethical clearance in Tanzania. All procedures were conducted in accordance with the Declaration of Helsinki and Good Clinical and Laboratory Practices. All participants gave informed written consent according to Good Clinical Practice guidelines.

### Material and methods

Women residing in Korogwe District, Tanga Region, Tanzania were followed throughout pregnancy as part of the observational cohort study STOPPAM (Strategies TO Prevent Pregnancy Associated Malaria). Pregnant women attending the Reproductive and Child Health (RCH) clinic at Korogwe District Hospital (KDH) or the Lwengera, Kerenge and Ngombezi Dispensaries were included in the study from September 2008 until March 2010. Follow-up was completed in October 2010.

Women with a gestational age (GA) of ≤24 weeks determined by ultrasound, having lived in Korogwe District for the past 6 months, willing to give birth at KDH and living in an accessible area were included in STOPPAM. The following conditions can affect fetal growth and BW and if present in the current pregnancy the woman/newborn was excluded from analysis; twin pregnancy [22], stillbirth, preterm delivery (GA<37 weeks) [8], multiple pregnancies, severe malformation [3], [22], maternal HIV infection [23], hypertensive disorders, malnutrition [3], diabetes, asthma, epilepsy, syphilis [24], severe anemia [25] and malaria [3], [5]. All conditions were diagnosed by the project team, except for maternal HIV and syphilis infection which were diagnosed by the governmental nurses and data extracted from the antenatal card, and asthma and epilepsy which were based on the medical history reported by the woman. Selection bias caused by an overrepresentation of women being willing to attend a RCH clinic early in pregnancy might have been introduced. Sensitization campaigns in the villages were performed to motivate all women to attend the RCH clinic and thereby reduce selection bias. The sample size for this study was not predefined, but determined by the STOPPAM projects core objective of evaluating the effect of malaria in pregnancy on the health of women and newborns requiring a sample size of 1000 women.

The women were followed at the RCH clinic at KDH at 3 pre-scheduled visits at a gestational week of 26, 30 and 36. If needed, women attended extra clinic visits. If women failed to attend the clinic, home visits were performed within one week of the booking.

At the first visit maternal age, obstetric history, chronic diseases, and socioeconomic status were documented. Throughout pregnancy the women were screened for the following conditions: malaria infection [positive blood smear and/or rapid diagnostic test (Parascreen™ Zephyr Biomedicals, Goa, India, Paracheck Orchid Biomedical Systems, Goa, India or ParaHIT Span diagnostics Ltd, Surat, India)], pregnancy induced hypertension (HT) [blood pressure ≥140 mmHg systolic and/or ≥90 mmHg diastolic (Digital machine, A&D Instruments, Japan and Spengler, France)] and preeclampsia [HT and ≥0,3 gr/L albumin on urine dipstick (SD Urocolour, Standard Diagnostics, Korea or Cybow, Cybow DFI, Korea)] both presenting after a GA of 20 weeks. HT diagnosed before a GA of 20 weeks was considered essential HT. Furthermore, diabetes defined as glucosuria followed by a random blood-glucose>11 mmol/L. It was considered gestational diabetes if the woman did not have pre-pregnancy diabetes. Malnutrition was defined as mid upper arm circumference<23 cm on inclusion [5] and severe anemia as a haemoglobin ≤7 g/dl (Sysmex hematological analyzer®, Kobe, Japan) at any time during pregnancy [25].

At delivery, birth weight (BW) (weighed naked, unadjusted for timespan since delivery) and sex were documented. Malformations were diagnosed with ultrasound during pregnancy or at birth. At the hospital, BW was measured using a spring scale (Fazzini®, Italy) to nearest 50 gr (until July 2009) or a digital strain gauge scale (ADE®, Germany) to nearest 10 gr (after July 2009). At home deliveries BW were measured using the Fazzini® spring scale. BW measured >24 hours after delivery were excluded from analyses [26], but FWs were still included from these newborns.

At the inclusion visit, GA was estimated using ultrasound and considered reliable until a GA of 24 weeks [27]. A new estimation was done within two months if the GA was <11 weeks at inclusion. The ultrasound based estimate was used to define GA for all women, and the GA was not changed at later visits. Variation in biometric measurements due to ethnic group is limited in early pregnancy, and biometric references for white populations were used [28], [29]. GA was estimated using crown-rump length (CRL) until at length of ≤75 mm (13 weeks and 4 days) and the Hadlock algorithm [29]. If CRL>75 mm head circumference (HC) was measured and converted according to Chitty *et al* [30]. HC is less affected by head shape and parity [28], [31] and was preferred to biparietal diameter.

At the visit at 26, 30 and 36 weeks of gestation HC, abdominal circumference (AC), and femur length (FL) were measured using techniques as described elsewhere [13] and recorded in millimeters. For each parameter, a mean of two measurements was used. If only one acceptable measurement was obtained a single measurement of the parameter was used. FWs were estimated (EFW) using the Hadlock algorithm [32]: Log10(EFW) = 1.326+0.0107*HC+0.0438*AC+0.158*FL – 0.00326*AC*FL. If it was not possible to obtain an acceptable HC, EFW was estimated using the Hadlock algorithm [32]: Log10(EFW) = 1.304+0.05281*AC+0.1938*FL – 0.004AC*FL.

To assess the accuracy of the Hadlock algorithm to predict FW in this population, BW estimates based on a projection of the last FW, assuming a weight gain of 24.2 g/day [33], was calculated (method A). For women with a FW measured within 35 days of delivery, BW was also estimated by applying the Hadlock proportionality formula [34], using the ratio between the individuals last EFW and the population median FW to predict the BW at term. As population reference the median FW and BW were extracted from the modified Hadlock chart developed using the method by Mikolajzcyk *et al* [20] (described below) (method B). BW had a non-parametric distribution and the estimated and the observed BW were compared using median error in grams and median percentage error. The percentage of BW estimates that were predicted accurately to within ±10% and ±15% of the observed BW was calculated [35]. The estimated BWs were only used for comparison and were not included in the development of the weight chart.

Ultrasound investigations were done at the RCH clinic at KDH by the first author and a local midwife trained for the study using a Sonosite TITAN®, US High resolution ultrasound system with a 5-2 MHz C60 abdominal probe. A few investigations were performed by a trained Tanzanian medical doctor. To evaluate and diminish inter-observer variability, randomly selected fetuses were measured by two investigators and measurements were compared. All investigations were stored as still pictures using SiteLink Image Manager 2.2.

Hybrid weight charts using a combination of EFW and observed BW were produced including only healthy pregnancies. The general weight chart was compared to the Congolese chart by Landis *et al* [10] and the chart by Hadlock *et al* [21]. Due to the closer geographic location of Congo to Tanzania, this chart was preferred over the chart from Burkina Faso [11]. The general weight chart was also compared to a modified Hadlock weigth chart using the web-based program by Mikolajzcyk *et al* [20]. The mean BW and variance (as a percentage) from newborns delivered at a GA of 40 to 40 weeks and 6 days in our cohort were imputed into the program. Using the ratio between the mean BW and the mean weight at term from the Hadlock chart the percentiles at all GA were calculated assuming a constant ratio and variance of the mean throughout pregnancy.

The prevalence of SGA in the cohort (weight below the 10^th^ percentile) [3], was evaluated by superimposing the observed BW on all the charts.

### Statistics

Data were double entered and validated using Microsoft Access 2007. Growth charts were developed using R 2011, and other statistical analyses performed in STATA 10. SigmaPlot 9.0 was used for graphical presentation.

The reference curves were constructed using local linear smoothing techniques [36] on a log-transformed version of the EFW/BW that lead to approximate normality of the residuals from the mean curve. The GA dependent variance was estimated using local linear smoothing of the squared residuals. Subsequently, we constructed the reference curves using the GA dependent mean and variance curves. This simple smoothing approach ignored the dependence in the repeated measurements within each subject, but in reality the smoothing primarily used independent measurements due to the somewhat regular pattern of the sampling ages for each woman. The bandwidth for the smoothing was selected by visual inspection. We further validated the results by random effect modeling using splines to fit the data. For this type of modeling the variance structure was derived from the specified random effects structure. The smoothing based technique and the random effects approach gave very similar results, but we preferred the simple non-parametric approach because of the full flexibility in mean and variance structure.

## Results

**Figure 1:**
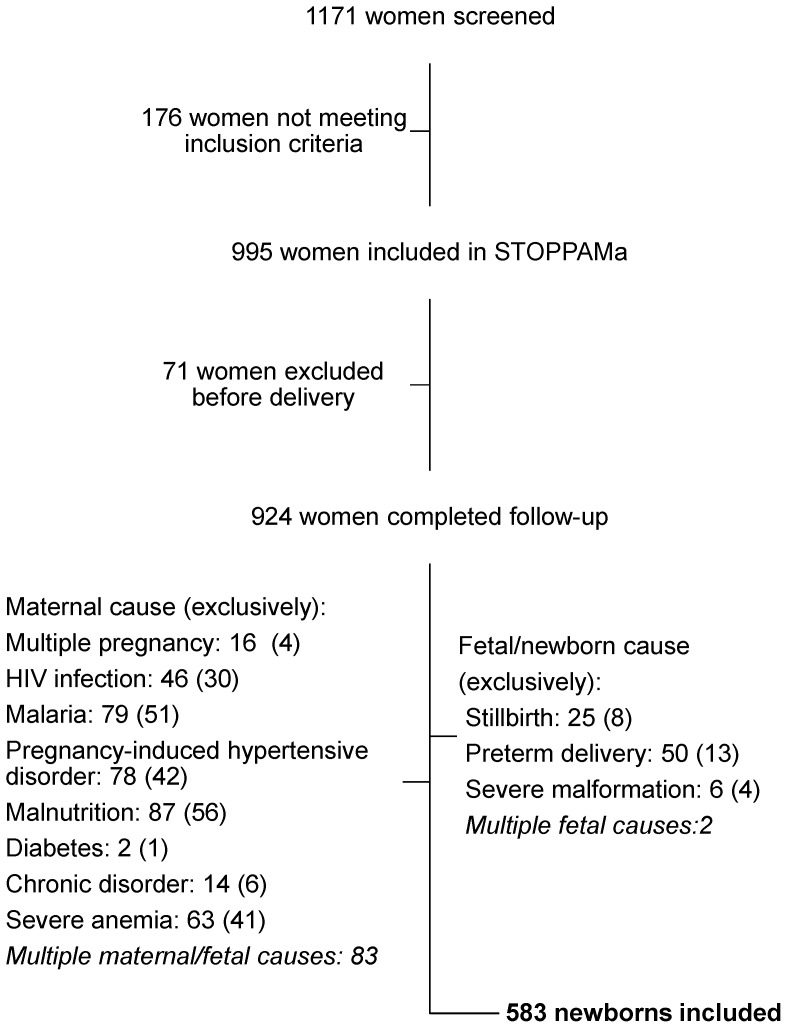
Flowchart of women/newborns included in the analyses. The number of women screened for inclusion and reasons for exclusion are shown. Of the 924 women/newborns completing follow-up 341 were excluded from analyses. Many suffered from multiple conditions. The number of women/newborns with a given conditions is stated. The numbers in brackets show women/newborns exclusively suffering from the given condition. Severe malformation includes omphalocele (2), hygroma (1), amyoplasia congenita athrygryposis (1), and large cystic abdominal process of unknown origin identified on ultrasound during pregnancy (2). Chronic disorders include asthma (10), epilepsy (1), syphilis (1), and essential hypertension (2). Diabetes includes diabetes mellitus (1) and gestational diabetes (1).

In total, 1171 pregnant women were screened and 995 met the inclusion criteria. Of these 21 women miscarried, 11 redrew consent, 5 moved out of the district, 34 was lost-to-follow-up and 924 women completed follow-up. Hereof 341 suffered from conditions possibly compromising fetal growth and BW (details shown in Figure 1), and 583 newborns remained for analyses. Characteristics of the included mothers and newborns are shown in Table 1.

**Table 1:** Characteristics of the 583 newborns included in the FW charts and their mothers.

Characteristic (unit) (N)		Mean ± SD (range)/% (N)
Maternal age (years) (582)		27.1±6.2 (15–47)
Ethnic group (582)	Sambaa	48.1 (280/582)
	Zigua	19.4 (113/582)
	Pare	7.0 (41/582)
	Bondei	3.8 (22/582)
	Other^a^	21.6 (126/582)
Maternal height (cm) (580)		157.6±5.9 (144–186)
Maternal weight (kg) (581)		55.7±9.4 (38–125.5)
Educational level (%) (579)	None	6.2 (36/579)
	Partial primary school	14.7 (85/579)
	Primary school compl.	66.1 (383/579)
	Second. school or above	13 (75(579)
Gravidity (%) (583)	Primigravidae	17 (99/583)
	Multigravidae	83 (484/583)
GA at inclusion (days) (583)		130^b^ (42–168)
Sex of newborn (%) (583)	Male	47.5 (277/583)
	Female	51.5 (300/583)
	Unknown	1(6/583)
Birth weight (g) (505)		3170^b^ (2040–4510)
GA at delivery (days) (581)		281^b^ (259–303)
Low birth weight^c^ (%) (505)		3.8 (19/505)

a ) Ethnic groups with less than 2% of the women represented.

b ) Median.

c ) BW<2500 g.

Code: GA = gestational age, G = gram, Kg = kilogram, N = number, SD = standard deviation.

The chart was developed based on 2193 weight measurement (1688 EFW and 505 BW) from the 583 newborns. Most fetuses (527/583∼90%) had three ultrasound derived weight measurements, 51 had two and five one. The mean interval between weight measurements was 33 days (SD±9.9, range: 6–84 days). The majority of FW were estimated at 26 (464 EFW), 30 (497 EFW) and 36 (491 EFW) weeks of gestation. Women showing early or late for their bookings had FW estimated slightly outside these time-points (35 EFW measured at week 24–25, 91 EFW at week 27–29, 84 EFW at week 31–35, and 26 EFW after week 36). Newborns with an available BW (505 BW) had a GA of 37–43 weeks at birth.

Ignoring negative sign the estimated BW compared to the observed BW had a median absolute prediction error of 228 g and 230 g and a percentage error of 6.8% and 7.5% for method A (505 BW) and method B (407 BW), respectively. When including negative sign the median prediction error was 94 g and 82 g. The estimated BW was within ±10% and ±15% of the observed BW for 68.1% and 84.8% of the newborns (method A), and for 63.9% and 82.3% of the newborns (method B), respectively. The median interval between last EFW and delivery were 27days and 25 days (method A and B, respectively).

**Figure 2:**
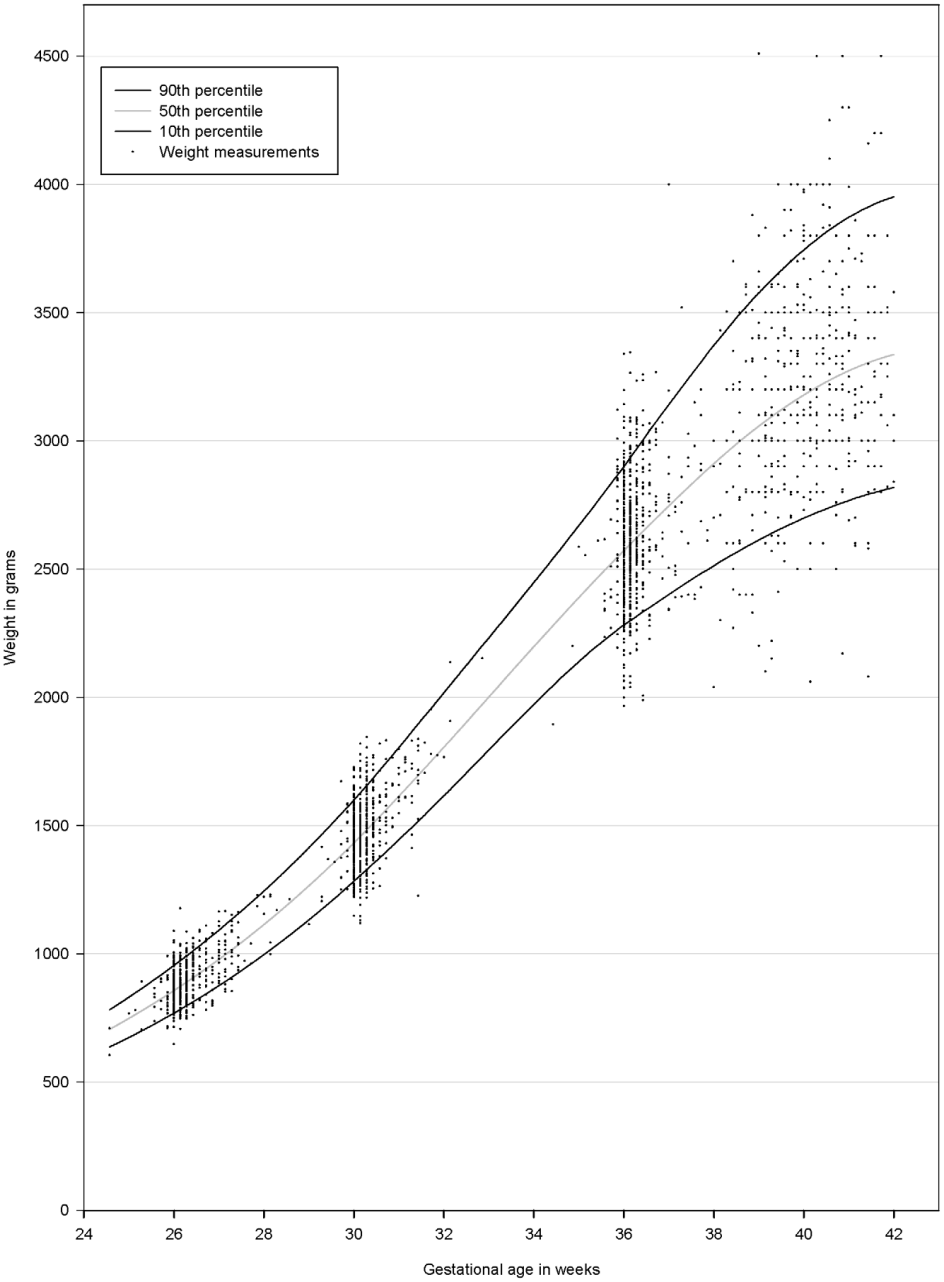
Fetal weight chart for the Tanzanian cohort. Both male and female fetuses/newborns are included. The 10^th^ (black —) 50^th^ (grey —) and 90^th^ (black —) percentiles are shown. The weight measurements for the individual fetuses/newborns are superimposed on the chart (^.^).

A general weight chart was produced and is presented in Figure 2 with the measured EFW and BW superimposed. The variance increased with increasing GA.

The percentiles for the chart are shown in Table 2. Of the weight measurements 9.9% was below the 10^th^ percentile, 79.9% between the 10^th^ and 90^th^ percentiles and 10.2% above the 90^th^ percentile. Sex-specific charts and percentiles are available as supplementary information (Figure S1 and Table S1).

**Table 2:** Weight percentiles for the weight chart.

Weight percentiles (g)					
GA	*10th*	*25th*	*50th*	*75th*	*90th*
*25*	674	708	748	791	831
*26*	769	809	857	907	955
*27*	876	923	978	1037	1093
*28*	997	1051	1114	1181	1245
*29*	1133	1194	1266	1342	1414
*30*	1283	1352	1433	1519	1600
*31*	1446	1524	1615	1712	1805
*32*	1617	1704	1806	1915	2018
*33*	1796	1890	2002	2120	2232
*34*	1973	2076	2198	2326	2448
*35*	2138	2254	2389	2533	2670
*36*	2283	2416	2572	2739	2899
*37*	2401	2559	2747	2948	3142
*38*	2513	2694	2912	3146	3373
*39*	2614	2815	3058	3321	3578
*40*	2700	2917	3179	3466	3745
*41*	2767	2997	3274	3577	3873
*42*	2818	3053	3337	3648	3952

Code: GA = Gestational age in weeks.

**Figure 3:**
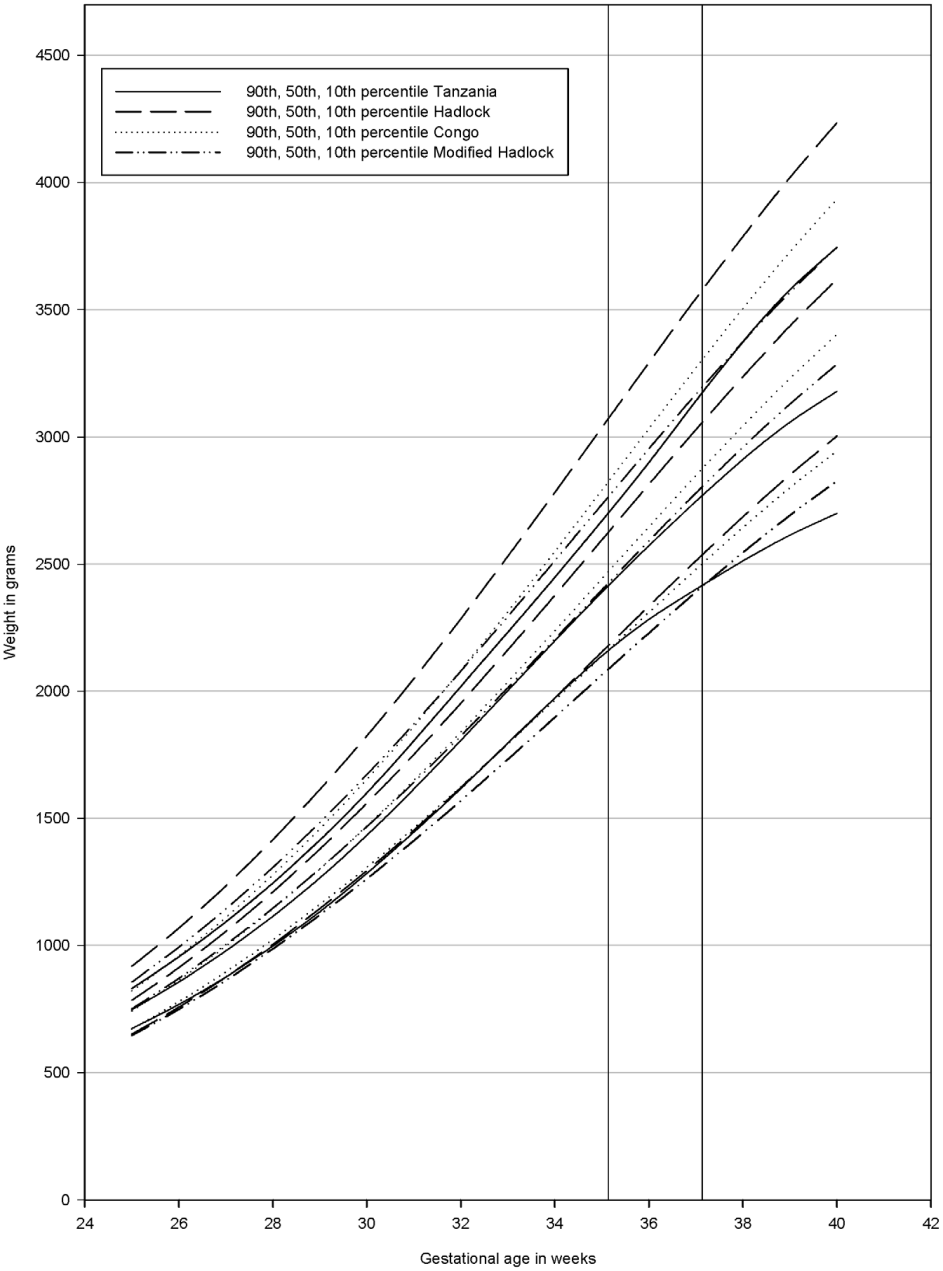
Comparison of the different fetal weight charts. Our Tanzanian chart (—), the Hadlock chart [21] (-----), the Congolese chart [10] (^…^), the chart developed using the method suggested by Mikolajzcyk *et al* [20] modifying the Hadlock chart using local birth weights(–^..^–). One vertical line crosses at a GA of 35 weeks and 1 day indicating when the Tanzanian chart's 10^th^ percentile deviates from the Congolese chart's and the Hadlock chart's 10^th^ percentiles. One vertical line crosses at a GA of 37 weeks and 1 day indicating when the Tanzanian cohorts' 10^th^ percentile deviates from the modified Hadlock chart's 10^th^ percentile.

The Congolese weight chart by Landis *et al* [10] and the weight chart produced in a white population by Hadlock *et al* [21], both based on ultrasound measurements, were compared to our chart (Figure 3).

The Hadlock weight chart modified by using the mean BW in our population [20] (Figure 3), was also compared to our chart. The modified Hadlock chart was developed based on 156 newborns with a mean BW of 3286 g with a constant variance of 12.3% of the mean at any given GA.

All three charts had percentiles at higher weights than what was observed in our chart. In particular, the 90^th^ and 50^th^ percentiles for the Congolese and the Hadlock charts deviate from our chart. The 10^th^ percentiles are similar until at a GA of 35 weeks and 1 day, but hereafter the Congolese chart and the Hadlock chart have percentiles at increasingly higher weights compared to ours. The modified Hadlock chart had a similar 50^th^ percentile until a GA of 36 weeks. Applying a constant variance in the modified Hadlock chart led to an increased difference from the percentiles of our chart at lower gestational ages for the 90^th^ and 10^th^ percentile. Furthermore, the modified Hadlock chart had a 10^th^ percentile at increasingly higher weights after a GA of 37 weeks and 1 day compared to our chart.

Percentiles from the Hadlock and the Congolese chart were available until a GA of 40 weeks [10], [21]. Among the newborns with a GA≤40 weeks (250/505) the prevalence of SGA was 28.8% (72), 22% (55) and 15.2% (38) using the Hadlock, the Congolese and the modified Hadlock weight charts as a reference, respectively. When applying our own chart it was 10.8% (27).

## Discussion

We produced a weight chart based on measurements of FW and term BWs. Ultrasound was not performed routinely after 37 weeks of gestation and we therefore opted for at hybrid FW chart as argued by others to be advantages in such a situation [37], [38]. The design of the study meant a clustering of FW estimations at 26, 30 and 36 weeks of gestation. The percentiles at weeks 27–29 and 31–35 should therefore be interpreted with cautions as the lack of data-points necessitated interpolation in these time intervals.

In order to produce a standard weight chart representing the optimal growth potential in this population we excluded women with pathology. This is in line with Zhang *et al* [19] and Mikolajczyk *et al* [20] who argued that standard weight charts based on healthy pregnancies have higher sensitivity in identifying SGA and better clinical utility than population weight charts including all pregnancies.

To access the accuracy of the Hadlock formula to predict FW we estimated BW both by using a predictive weight gain of 24.2 g/day [33] and the proportionality formula described by Mongelli *et al* [34]. FW gain estimates have not been reported for African populations, and we therefore used an estimate originating from a white population to predict BW from EFW. Estimated and observed BWs were in good agreement with 84.8% and 82.3% of the estimated BWs being within ±15% of the observed BW using the two methods. This is comparable with other studies [35], [39]. On average estimated BW slightly overestimated the observed BW.

The overestimation could be explained by a tendency of overestimating FW when using the Hadlock formula in an African population as reported by Mirghani *et al* [39]. Various studies find an effect of ethnicity on biometric measurements in the last half of pregnancy [13]–[16]. The Hadlock formula is developed on biometric measurements on a white population and on an assumption of fixed proportions between the biometric measurements. A shift in biometric proportions could affect the EFW calculated using the Hadlock formula. Currently, there is no weight formula developed on an African population. However, with an average percentage error of only 6.8% and 7.5% we believe the produced weight chart is valid.

The slight tendency of the Hadlock formula to overestimate the EFW could explain the observed flattening of the percentiles in the end of pregnancy. It might also reflect a true decrease in growth velocity. Studies among Peruvian [18] and Mexian [17] women also indicated a slower growth rate in end pregnancy. A decrease in growth velocity has also observed in a white population [40], and declining growth velocity in term pregnancies might be present in many populations in varying degrees.

Our chart differed from all charts used for comparison [10], [20], [21] leading to a substantial overestimation of the prevalence of SGA, when these charts were used. Our study used the Hadlock algorithm based on HC, FL and AC to estimate FW, whereas the other studies used the Hadlock algorithm based on HC, BPD, FL and AC [32]. The algorithms should be compatible in the ability to estimate FW [32] and are some of the most accurate [35], [39].

The fact that the Congolese differed is interesting. Landis *et al* [10] included women suffering from malaria, malnutrition and obstetrical complications. When we produced weight charts using the same inclusion criteria as Landis *et al* [10] the difference was a bit more pronounced (data not shown). The difference between the Congolese and the Tanzanian weight chart could also be due to differences in fetal growth patterns or socioeconomic status. The study by Landis *et al* [10] was conducted in an urban population whereas Korogwe district is a mixture of a semi-urban and rural population. Finally, the observed difference could be due to constitutional differences, and a statistically significant difference in maternal height in the two studies was observed (data not shown). Recently, Gaillard *et al* [41] customized FW charts by maternal and fetal characteristics applying the method developed by Gardosi *et al* [42]. Gaillard *et al* showed that maternal and fetal characteristics modulate the FW chart throughout the 3^rd^ trimester. The purpose of customization is to differentiate the growth retarded offspring from the constitutional small (SGA) but healthy offspring. Customization of the charts might reveal that part of the differences is explained by constitutional differences. Customized weight charts have in some studies proved superior to populations based charts in diagnosing SGA and predicting poor birth outcomes [4], [43]. Others argue that customisation only identifies a small additional group of only moderate increased risk of morbidity and mortality [44], [45].

However, the difference between the Congolese and our chart emphasizes the importance of producing regional specific or even country specific standard FW charts.

In Tanzania the majority of the ethnic groups originate from the Bantu people and in Korogwe District the dominating groups are Sambaa and Zigua. Other Bantu groups are also represented due to considerable internal movement in Tanzania [46]. In our study 32.5% belonged to other ethnic groups than Sambaa and Zigua, and most groups accounted for less than 2% of the women. Paternal ethnicity was not known. Therefore, we could not with certainty determine the newborns ethnicity and weight charts for ethnic sub-groups were not developed. There could be regional differences within Tanzania in FW, but due to the ethnic diversity in our study population we believe our chart can be considered representative for Tanzania.

Mikolajczyk *et al* [20] suggested to adapt the Hadlock chart [21] using the actual mean BW at term for local populations. This approach does not take into account the possible difference in growth velocity between populations [17], [18]. When comparing our chart with the modified Hadlock chart the difference increased at the end of pregnancy indicating a different growth pattern in our population. Furthermore, the variance of weights increased as GA increased. The method suggested by Mikolajczyk *et al* [20] assumes a constant variance throughout pregnancy leading to a too large variance in early gestations, and possibly leading to inaccurate classification of SGA. However the modified Hadlock chart is easy to use, can be applied worldwide, and was the one most similar to our chart. With this study we confirm that even though it is not perfect, it is a good alternative if an ultrasound derived weight chart is not available.

In conclusion, we present a weight chart to be used for clinical evaluation of the progress of pregnancies in a resource-poor setting in Tanzania. Furthermore, we demonstrate the large difference in fetal growth in Africa populations. This is an important message for all clinicians and researchers working in Africa.

Many studies evaluating the effect of various exposures on fetal growth are being conducted in Tanzania. Birth weights are compared to weight charts from white populations as a proxy measure for compromised fetal growth [5]. This chart will function as an important research tool for a more accurate evaluation of the effect of various exposures on fetal growth. Many regions in the developed world are still without weight charts and more investment in this field is needed. In the absence of ultrasound derived fetal weights the modified Hadlock chart suggested by Mikolajczyk *et al* [20] provides an acceptable alternative.

## Supporting Information

Figure S1 **FW charts for the Tanzanian female and male cohort presented separately.** Female percentiles (-----) and male percentiles (—) are shown. The sex-specific FW charts were based on 300 female newborns with 1139 weight measurements and 277 male newborns with 1037 weight measurements. Of the weight measurements 10.4% was below the 10^th^ percentile, 79.6% between the 10^th^ and 90^th^ percentiles and 10.0% above the 90^th^ percentile for the female chart. For the male chart the distribution was 9.5%, 80.0%, 10.5% below the 10^th^, between the 10^th^–90^th^, and above the 90^th^ percentile, respectively. Until a GA of 38 weeks and 6 days the sex-specific charts are similar. Thereafter, the males have higher weights than the females. The vertical line indicates when the female's percentiles deviate from the male's percentiles.
(TIF)

Table S1 Weight percentiles for the female and male sex-specific weight charts.
(DOC)
