# Supplementary figures and images for: Development of a Fetal Weight Chart Using Serial Trans-Abdominal Ultrasound in an East African Population: A Longitudinal Observational Study
Source: PLoS One. 2012 Sep 21;7(9):e44773. doi: 10.1371/journal.pone.0044773 (PMC3448622; doi:10.1371/journal.pone.0044773)

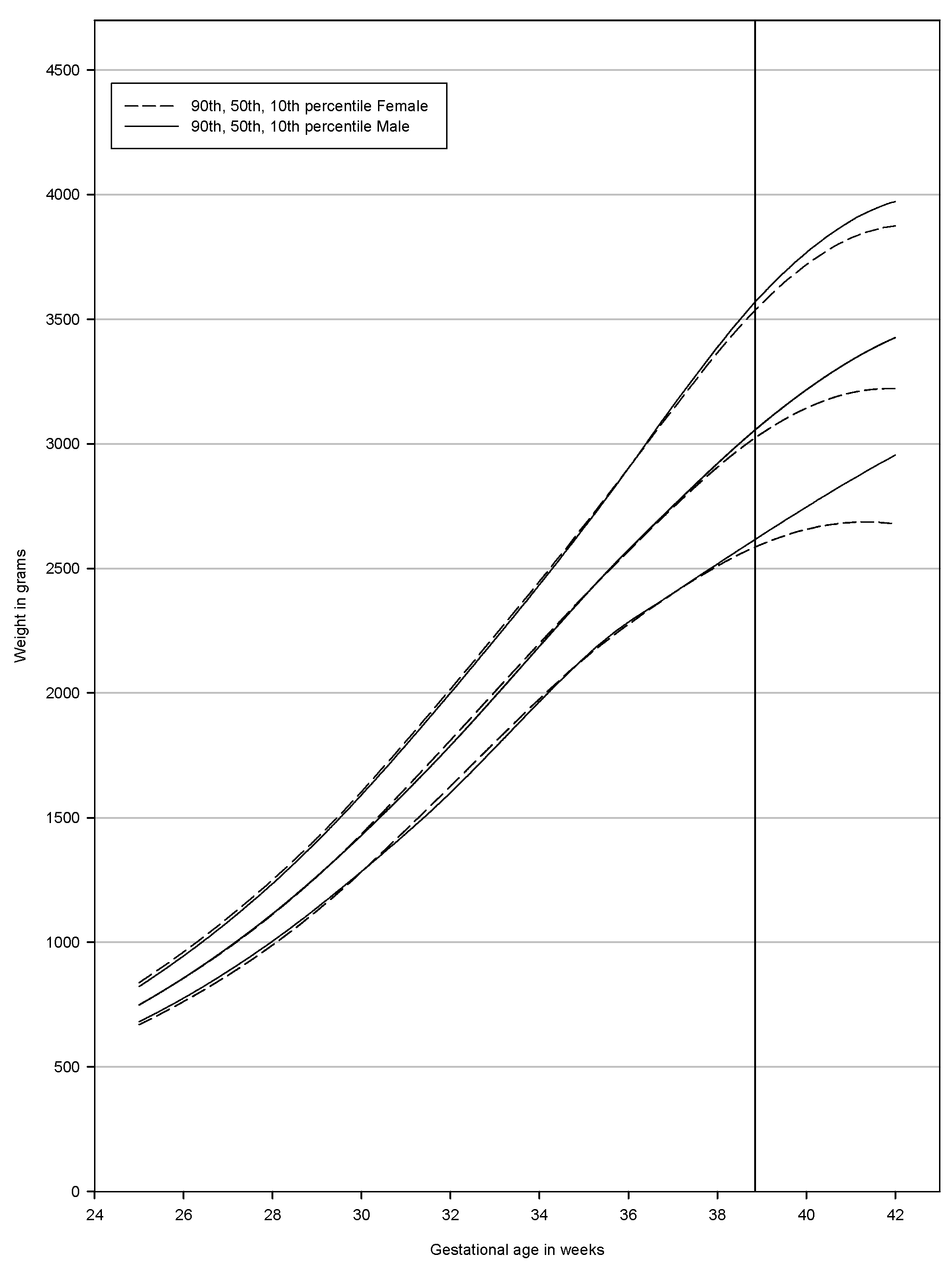

Supplement: Figure S1 — FW charts for the Tanzanian female and male cohort presented separately. Female percentiles (-----) and male percentiles (—) are shown. The sex-specific FW charts were based on 300 female newborns with 1139 weight measurements and 277 male newborns with 1037 weight measurements. Of the weight measurements 10.4% was below the 10th percentile, 79.6% between the 10th and 90th percentiles and 10.0% above the 90th percentile for the female chart. For the male chart the distribution was 9.5%, 80.0%, 10.5% below the 10th, between the 10th–90th, and above the 90th percentile, respectively. Until a GA of 38 weeks and 6 days the sex-specific charts are similar. Thereafter, the males have higher weights than the females. The vertical line indicates when the female's percentiles deviate from the male's percentiles. (TIF) [file pone.0044773.s001.tif]
