# Supplementary material for: Development of a Fetal Weight Chart Using Serial Trans-Abdominal Ultrasound in an East African Population: A Longitudinal Observational Study
Source: PLoS One. 2012 Sep 21;7(9):e44773. doi: 10.1371/journal.pone.0044773 (PMC3448622; doi:10.1371/journal.pone.0044773)
Supplement: Table S1 — Weight percentiles for the female and male sex-specific weight charts. (DOC) [file pone.0044773.s002.doc]

**Supplementary Table SI**. Weight percentiles for the female and male sex-specific weight charts.

| Weight percentiles (g) | | | | | | | | | | |
| --- | --- | --- | --- | --- | --- | --- | --- | --- | --- | --- |
|  | Female | | | | | Male | | | | |
| GA* | *10th* | *25th* | *50th* | *75th* | *90th* | *10th* | *25th* | *50th* | *75th* | *90th* |
| *25* | 670 | 706 | 749 | 795 | 839 | 681 | 713 | 749 | 787 | 823 |
| *26* | 763 | 806 | 856 | 910 | 962 | 777 | 814 | 857 | 903 | 946 |
| *27* | 868 | 918 | 977 | 1040 | 1099 | 885 | 928 | 979 | 1032 | 1083 |
| *28* | 989 | 1046 | 1112 | 1183 | 1251 | 1005 | 1055 | 1114 | 1177 | 1236 |
| *29* | 1127 | 1190 | 1264 | 1344 | 1419 | 1139 | 1197 | 1265 | 1337 | 1405 |
| *30* | 1283 | 1353 | 1453 | 1522 | 1605 | 1285 | 1351 | 1430 | 1513 | 1592 |
| *31* | 1452 | 1529 | 1620 | 1716 | 1807 | 1437 | 1514 | 1605 | 1701 | 1792 |
| *32* | 1627 | 1712 | 1811 | 1916 | 2016 | 1601 | 1688 | 1790 | 1898 | 2001 |
| *33* | 1803 | 1897 | 2006 | 2121 | 2231 | 1780 | 1875 | 1986 | 2104 | 2216 |
| *34* | 1976 | 2079 | 2200 | 2327 | 2449 | 1965 | 2068 | 2188 | 2315 | 2435 |
| *35* | 2137 | 2253 | 2389 | 2534 | 2672 | 2140 | 2254 | 2387 | 2529 | 2663 |
| *36* | 2276 | 2411 | 2570 | 2740 | 2903 | 2285 | 2418 | 2575 | 2742 | 2902 |
| *37* | 2401 | 2558 | 2744 | 2945 | 3137 | 2400 | 2560 | 2751 | 2955 | 3152 |
| *38* | 2510 | 2691 | 2907 | 3140 | 3366 | 2518 | 2702 | 2921 | 3158 | 3388 |
| *39* | 2598 | 2800 | 3043 | 3307 | 3564 | 2634 | 2836 | 3079 | 3342 | 3598 |
| *40* | 2657 | 2878 | 3144 | 3435 | 3719 | 2747 | 2960 | 3217 | 3496 | 3768 |
| *41* | 2685 | 2920 | 3205 | 3519 | 3827 | 2855 | 3073 | 3334 | 3619 | 3895 |
| *42* | 2680 | 2924 | 3222 | 3551 | 3875 | 2956 | 3170 | 3427 | 3704 | 3973 |

Code: GA = Gestational age in weeks
